# Research Trends in Immune Checkpoint Blockade for Melanoma: Visualization and Bibliometric Analysis

**DOI:** 10.2196/32728

**Published:** 2022-06-27

**Authors:** Yantao Xu, Zixi Jiang, Xinwei Kuang, Xiang Chen, Hong Liu

**Affiliations:** 1 Department of Dermatology Xiangya Hospital Central South University Changsha China; 2 National Engineering Research Center of Personalized Diagnostic and Therapeutic Technology Xiangya Hospital Central South University Changsha China; 3 Hunan Engineering Research Center of Skin Health and Disease Xiangya Hospital Central South University Changsha China; 4 Hunan Key Laboratory of Skin Cancer and Psoriasis Xiangya Hospital Central South University Changsha China; 5 Xiangya School of Medicine Central South University Changsha China; 6 National Clinical Research Center of Geriatric Disorders Xiangya Hospital Central South University Changsha China

**Keywords:** melanoma, immune checkpoint blockade, bibliometric, research trends, dermatology, cancer

## Abstract

**Background:**

Melanoma is one of the most life-threatening skin cancers; immune checkpoint blockade is widely used in the treatment of melanoma because of its remarkable efficacy.

**Objective:**

This study aimed to conduct a comprehensive bibliometric analysis of research conducted in recent decades on immune checkpoint blockade for melanoma, while exploring research trends and public interest in this topic.

**Methods:**

We summarized the articles in the Web of Science Core Collection on immune checkpoint blockade for melanoma in each year from 1999 to 2020. The R package bibliometrix was used for data extraction and visualization of the distribution of publication year and the top 10 core authors. Keyword citation burst analysis and cocitation networks were calculated with CiteSpace. A Gunn online world map was used to evaluate distribution by country and region. Ranking was performed using the Standard Competition Ranking method. Coauthorship analysis and co-occurrence were analyzed and visualized with VOSviewer.

**Results:**

After removing duplicates, a total of 9169 publications were included. The distribution of publications by year showed that the number of publications rose sharply from 2015 onwards and either reached a peak in 2020 or has yet to reach a peak. The geographical distribution indicated that there was a large gap between the number of publications in the United States and other countries. The coauthorship analysis showed that the 149 top institutions were grouped into 8 clusters, each covering approximately a single country, suggesting that international cooperation among institutions should be strengthened. The core author extraction revealed changes in the most prolific authors. The keyword analysis revealed clustering and top citation bursts. The cocitation analysis of references from 2010 to 2020 revealed the number of citations and the centrality of the top articles.

**Conclusions:**

This study revealed trends in research and public interest in immune checkpoint blockade for melanoma. Our findings suggest that the field is growing rapidly, has several core authors, and that the United States is taking the lead position. Moreover, cooperation between countries should be strengthened, and future research hot spots might focus on deeper exploration of drug mechanisms, prediction of treatment efficacy, prediction of adverse events, and new modes of administration, such as combination therapy, which may pave the way for further research.

## Introduction

In the past 10 years, although the frequency of melanoma has continued to increase, the lethality of advanced melanoma has decreased. Nevertheless, melanoma is still one of the most life-threatening skin cancers [1]. Globally, melanoma cases have grown rapidly. The incidence of new melanomas exceeded 350,000 in 2015 [2]; moreover, the rate of new melanomas has continually increased for the last 20 years [3]. Immune checkpoint blockade (ICB) is widely used in the treatment of melanomas, especially those with negative regulators, such as T-lymphocyte associated protein-4 (CTLA-4), programmed death receptor 1 (PD-1), and the ligands of PD-1 (PD-L1). ICBs are often chosen as the subject of clinical trials. The reason for the remarkable efficacy of ICBs in melanoma is the biological features of the cancer. Melanoma is often described as an archetypal immunogenic cancer, which guarantees the efficacy of immunotherapy; this is supported by many studies that have observed tumor progression and increased rates of melanoma in immunosuppressed individuals [4,5]. Moreover, melanoma always carries a large tumor mutation burden [6], which increases the probability of driving out a stronger immune response in the host. Because of these biological and clinical features, ICBs for melanoma have been thoroughly researched in the past two decades and have gradually became a research hotspot.

Bibliometric analysis is a quantitative science approach using methods such as co-occurrence analysis and citation analysis to evaluate research performance [7,8]. In the health care field, bibliometrics are mostly used to measure the influence or impact of research articles. Bibliometric methods estimate how much influence or impact a selected research article may have on future research, and the results are especially valuable for those topics that are gradually becoming more intriguing. However, there has been no bibliometric analysis of ICB for melanoma. In this study, we conducted a comprehensive bibliometric analysis covering recent decades while exploring research trends and the public interest in ICB for melanoma. We determined the research landscape of ICB for melanoma in terms of chronological distribution, geographical distribution, publication sources, author publications, and cocitations. We also identified the co-occurrence of authors, organizations, and keywords. The purpose of this study is to provide a systematic summary of research trends and the public interest in ICB for melanoma from an evaluative bibliometric perspective.

## Methods

### Data Sources and Search Strategy

Bibliographic data for the analysis were all acquired from the Web of Science Core Collection, which includes the Science Citation Index Expanded, Social Science Citation Index, and Emerging Source Citation Index [9]. To perform a comprehensive literature search of immune checkpoint inhibitors in melanoma, we designed a systematic search strategy. Generally, the search strategy was as follows: (TS=(ipilimumab OR pembrolizumab OR nivolumab OR immunotherapy OR “immune checkpoint blockade” OR PD-1 OR PD-L1 OR CTLA-4) OR TI=(ipilimumab OR pembrolizumab OR nivolumab OR immunotherapy OR “immune checkpoint blockade” OR PD-1 OR PD-L1 OR CTLA-4 OR yervoy OR Keytruda OR opdivo) OR AB=(ipilimumab OR pembrolizumab OR nivolumab OR immunotherapy OR “immune checkpoint blockade” OR PD-1 OR PD-L1 OR CTLA-4 OR yervoy OR Keytruda OR opdivo)) AND (TS=(melanoma OR melanocarcinoma) OR TI=(melano* OR melanoma OR melanocarcinoma) OR AB=(melano* OR melanoma OR melanocarcinoma)). The language was restricted to English and the document type was limited to articles and reviews. The time span of the search excluded the year 2022 for clearer annual results.

**Figure 1:**
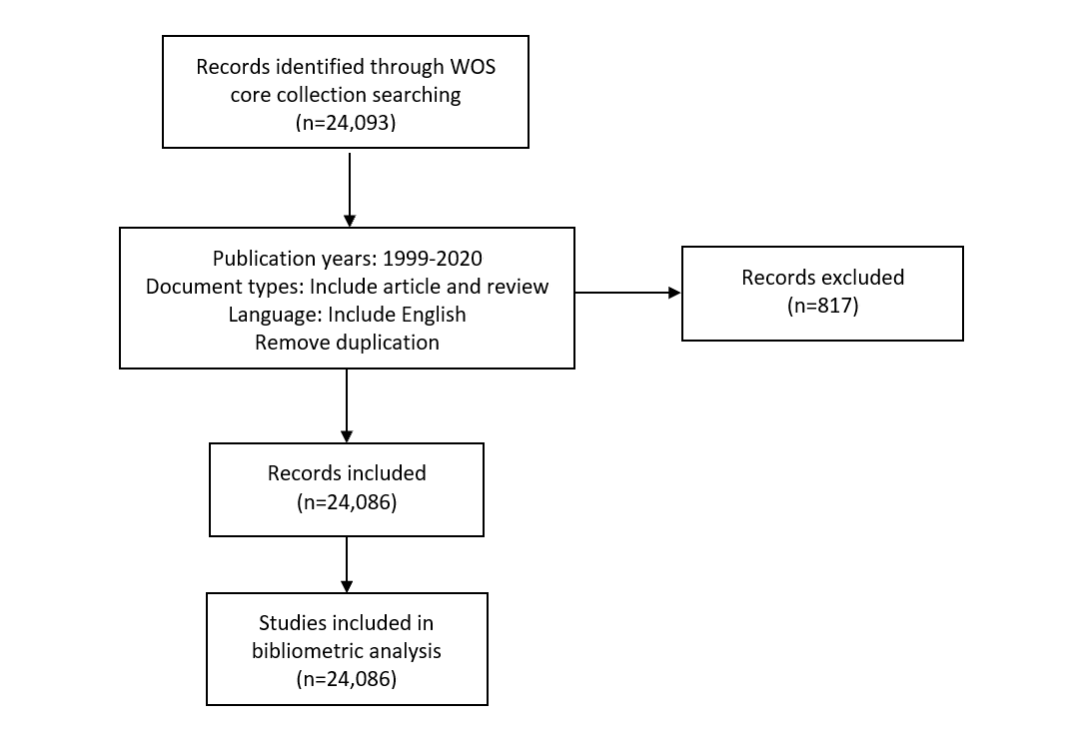
Detailed search flowchart, showing steps in the identification and screening of papers. Publication years spanned 1999 to 2021. Only documents published in English were included. Endnote was used to remove duplicates. The R package Bibliometrix was used to remove documents that were published as preprints in 2021 by extracting the publication date.

A total of 24,093 documents were retrieved from the Web of Science Core Collection. After excluding documents that were published as preprints in 2021 and then published as final versions in 2022 and documents with an unknown publication date, 24,086 documents remained in the bibliometric analysis and visualization. The search details are presented as a flowchart (Figure 1). The search was completed on December 6, 2021.

### Data Extraction and Analysis

The retrieval characteristics used for publications on ICB for melanoma included the distribution of publication year, country and region, organization, journal, core authors, keywords, and key references. The detailed search strategy is shown in Multimedia Appendix 1. Bibliometric analysis and network visualization were performed with VOSviewer (version 1.6.14, Leiden University), CiteSpace (version 5.7.R5W, Drexel University), and the bibliometrix package in R (version 3.6, R Foundation). The bibliometrix package was used for data extraction and visualization of the distribution of publication year and the top 10 core authors. The keywords citation burst analysis and cocitation network analysis were performed with CiteSpace. The Gunn online world map was used to evaluate the distribution of countries and regions. Ranking was performed using the standard competition ranking method. Other analyses, including the coauthorship analysis, co-occurrence analysis, and visualization, were conducted with VOSviewer.

## Results

### Distribution of Publications

**Figure 2:**
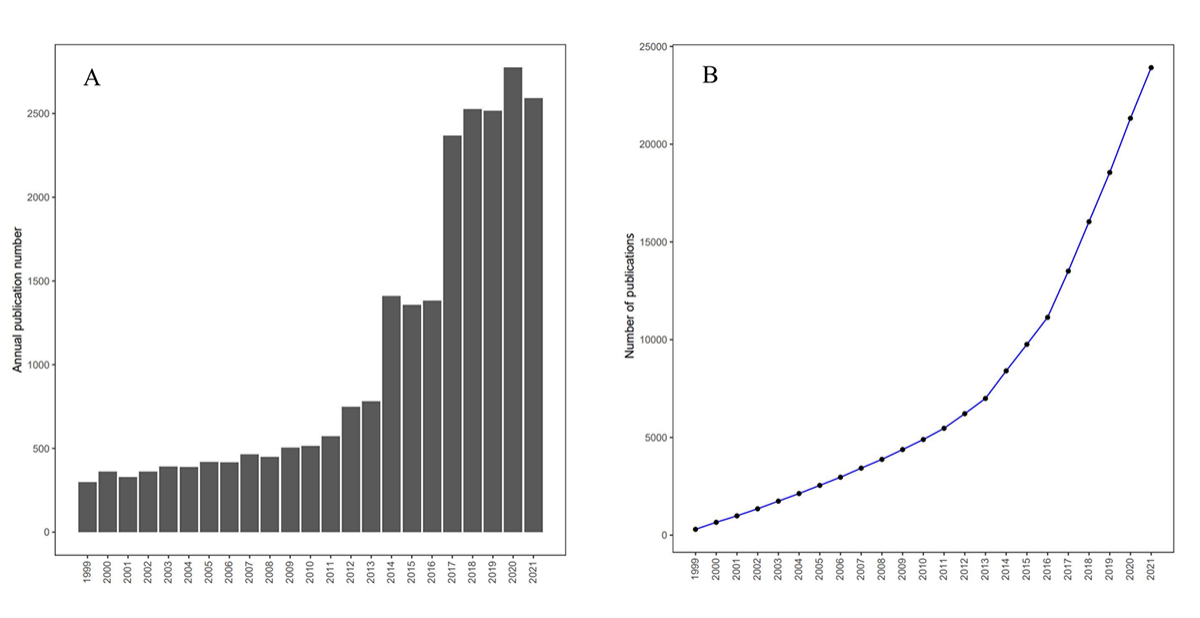
Distribution of publications by year. (A) The cumulative number of publications and (B) the annual number of publications on immune checkpoint blockade for melanoma. The peak of cumulative publications occurred in 2020. The annual number of publications increased relatively slowly from 1999 to 2021 and sharply from 2014 to 2017 and onwards. The peak of annual publication either occurred in 2020 or has yet to occur. The publication data for 2021 does not include data for December.

Figure 2 shows the chronological distribution of publications by year as a bar chart (Figure 2A). From 1999 to 2013, the annual number of publications steadily grew, with no obvious research trends, and remained relatively stable. The annual number of publications then rose sharply from 2015 to 2020. Figure 2B shows the total, cumulative number of publications as a plot. There was a relatively slow increase in the cumulative number of publications from 1999 to 2015, with the number of publications growing sharply from 2015 to 2017 onwards; the peak either occurred in 2020 or has yet to occur.

**Figure 3:**
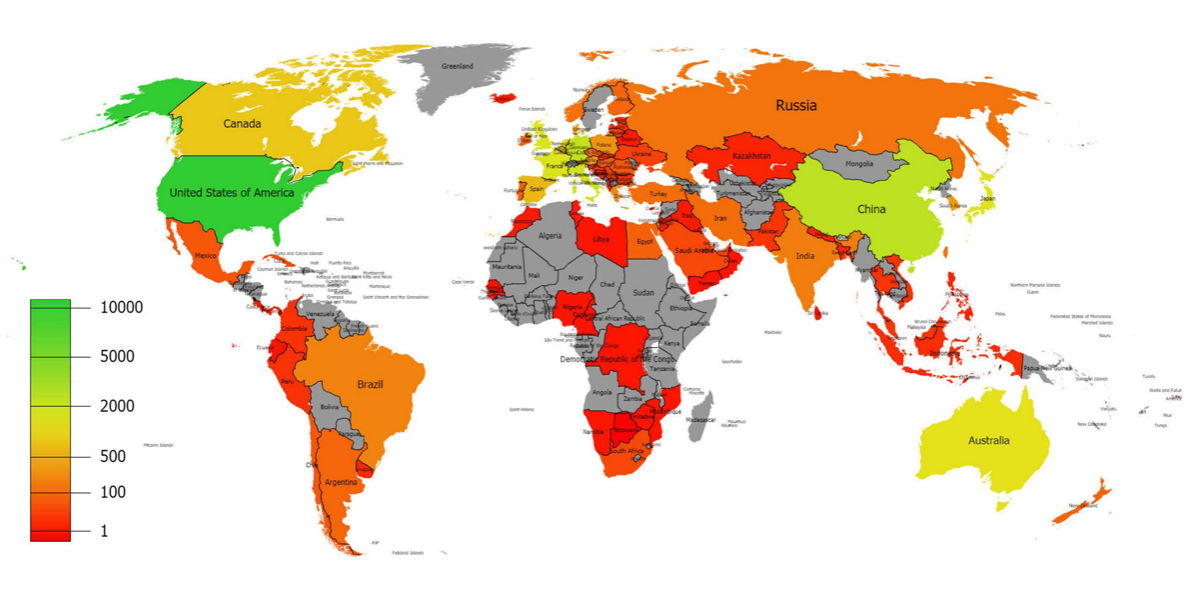
Geographical distribution of global publications. The green-to-red gradient represents a decreasing number of publications. Gray represents countries with no publications.

As for geographical distribution, 24,086 documents were published from 117 different countries and regions. Studies involving multiple countries were included in the analysis, with each country being counted individually. We classified documents by country and visualized the spatial distribution as a heatmap (Figure 3). Table 1 lists the top 12 most prolific countries. In total, the country with the largest number of publications was the United States (11,113/24,086 publications, 46.1%), far surpassing China (2345/24,086 publications, 9.7%) and Germany (2223/24,086 publications, 9.2%). As for citations, America was also far ahead. It is interesting that although China had the second largest number of publications, the number of citations lagged far behind other countries, which made the number of citations per publication rather small.

**Table 1:** Top 12 most productive countries and regions.

Rank	Country/region	Publications	Citations	Citations per publication
1	United States	11,113	642,788	57.84
2	China (mainland)	2345	45,215	19.28
3	Germany	2223	129,248	58.14
4	Italy	1847	87,903	47.59
5	France	1601	128,827	80.47
6	Japan	1598	53,323	33.37
7	England	1464	97,981	66.93
8	Australia	1381	81,059	58.70
9	Netherland	1087	75,699	69.64
10	Switzerland	945	50,695	53.64
11	Canada	862	77,164	89.52
12	Spain	658	58,543	88.97

### Analysis of Leading Organizations and Public Sources

**Figure 4:**
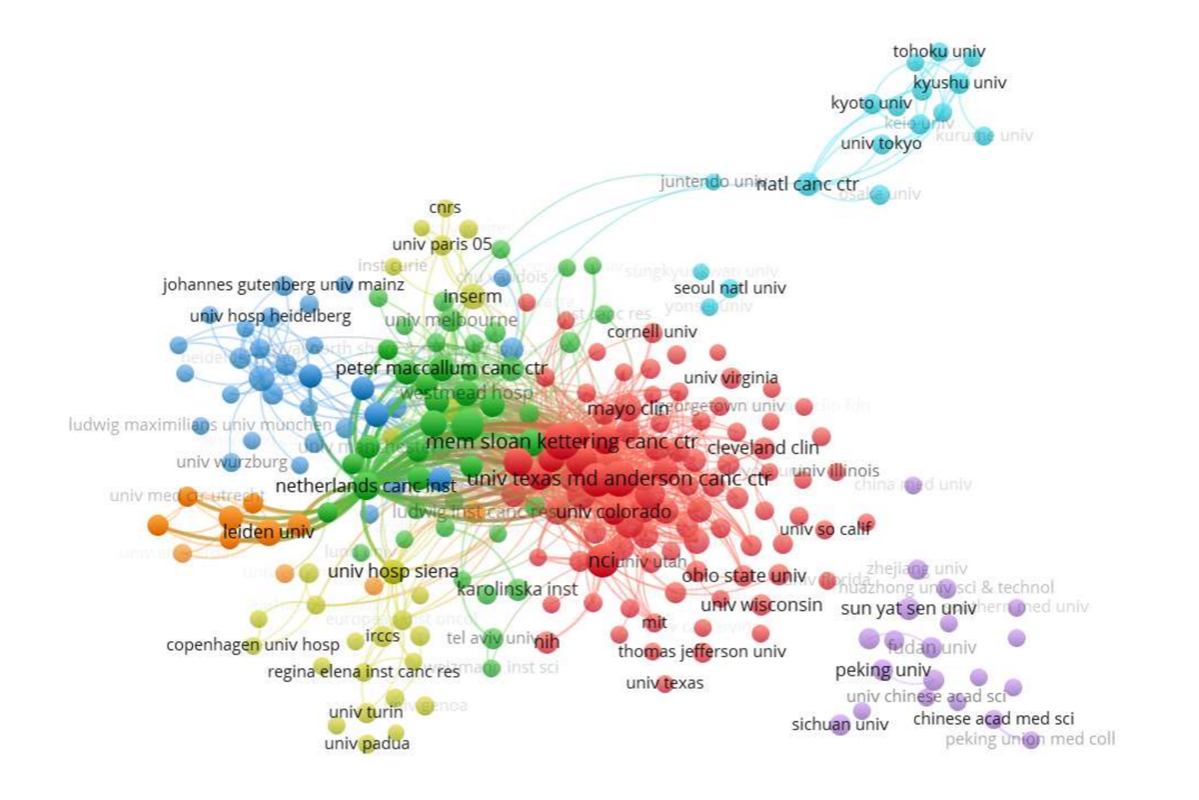
Coauthorship analysis of organizations. Plot showing a coauthorship analysis of organizations. The normalization method was fractionalization. The weight was the number of publications. The thickness of the lines indicates the strength of coauthorship relationships. Different colors indicate clusters.

The information on leading organizations was analyzed with VOSviewer. Generally, 24,086 documents were published by 13,359 different organizations. After merging duplicates and excluding disjointed organizations, a final total of 243 organizations met the inclusion threshold and are shown in the visualization. The top 10 most productive organizations are listed in Table 2. The most prolific organization was the Memorial Sloan Kettering Cancer Center (903/24,086 publications, 3.7%), followed by the University of Texas MD Anderson Cancer Center (859/24,086 publications, 3.6%) and the National Cancer Institute (645/24,086 publications, 2.7%). Among the top 10 institutions, 9 of 10 were from the United States, which corresponded to the distribution by country and region. We also conducted a coauthorship analysis of the organizations (Figure 4). We found that all 243 top published institutions were grouped into clusters, with each cluster representing approximately one country, except for the United States, which dominated 2 clusters and had wide correlations. The red and lower yellow clusters mainly represent American institutions, including Memorial Sloan Kettering Cancer Center, the MD Anderson Cancer Center, and the National Cancer Institution. The blue cluster represents Johannes Gutenberg University, in Germany. The upper yellow cluster represents universities and institutions in France. The green cluster includes other European countries and Australia. Two rather further away clusters mainly represent Japan and Korea, in light blue, and China, in purple, with few links with other clusters. This result suggests that the United States apparently led this topic and that international cooperation among various institutions from different countries should be strengthened, especially for institutions in China, Japan, and Korea.

**Table 2:** Top 10 most productive organizations.

Rank	Organization	Country	Articles	Citations	Total link strength^a^
1	Memorial Sloan Kettering Cancer Center	United States	903	120,565	3183
2	University of Texas MD Anderson Cancer Center	United States	859	54,089	2381
3	National Cancer Institute	United States	645	71,055	759
4	Dana-Farber Cancer Institute	United States	617	82,093	3079
5	University of Sydney	Australia	537	33,668	2749
6	University of Pittsburgh	United States	505	25,886	1310
7	Harvard Medical School	United States	480	21,477	1317
8	University of California Los Angeles	United States	476	50,391	1926
9	Massachusetts General Hospital	United States	440	32,663	1634
10	Mayo Clinic	United States	367	27,207	823

^a^Total link strength in VOSviewer represents all links between a given node and other nodes, which indicates how the entry interacts with other entries. The strength of a link is given by a nonnegative number. If one node has no links with other nodes, the total strength of the link equals zero.

Core journals were identified by analysis of publication sources. After analyzing bibliographies, we extracted the top 10 most prolific journals along with their impact factor (IF) in 2020 and 2021 in the field of ICB for melanoma (Table 3). We found that the most prolific journal was the *Journal of Clinical Oncology* (impact factor 44.544), which ranked first, with 1051 documents published. The second and the third most-prolific journals were *Cancer Immunology,* *Immunotherapy* (677 publications, IF 6.958) and *Cancer Research* (627 publications, IF 12.701). IF for the top 10 most prolific journals in 2019 ranged from 4.456 for the *Journal of Immunology* to 44.544 for the *Journal of Oncology*. Our findings for the total number of publications and IF suggest that *Cancer Research* might be the most influential journal in the field of ICB for melanoma.

**Table 3:** Top 10 most prolific journals.

Rank	Journal	Publications	Impact factor (2020)	Impact factor (2021)
1	*Journal of Clinical Oncology*	1051	32.956	44.544
2	*Cancer Immunology, Immunotherapy*	677	5.442	6.958
3	*Cancer Research*	627	9.727	12.701
4	*Clinical Cancer Research*	602	10.107	12.531
5	*Journal for Immunotherapy of Cancer*	589	9.913	13.751
6	*Oncoimmunology*	508	5.869	8.11
7	*Annals of Oncology*	498	18.274	32.976
8	*Journal of Immunotherapy*	465	4.11	4.456
9	*Journal of Immunology*	359	4.886	5.422
10	*Frontiers in Immunology*	336	5.085	7.561

### Analysis of Coauthorship and Core Authors

**Figure 5:**
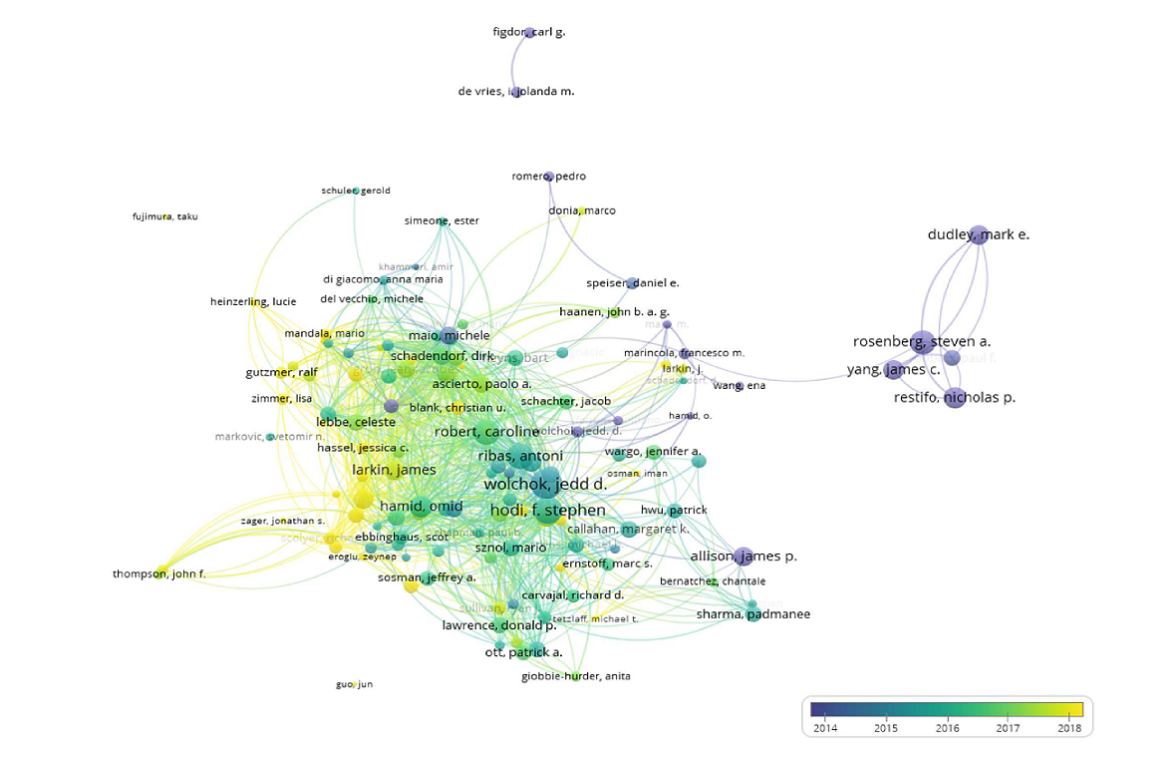
Overlay visualization of coauthorship relationships between authors. The analysis method was Linlog/modularity. The weight was citations. Scores are the average year of publication. The thickness of the lines indicates the strength of the relationships. The colors of the circles represent the average year of publication.

Information on authors and co-authors was also analyzed with VOSviewer. A total of 24,086 publications were produced by a total of 93,587 authors. The 3 most important evaluation criteria for core authors included the number of published documents, total citations, and the H index. Therefore, we extracted and visualized the top 10 most prolific authors according to these criteria (Table 4 and Figure 5). Paolo A Ascierto, the director of the Unit of Melanoma, Cancer Immunotherapy and Innovative Therapy at the National Tumour Institute, Fondazione G. Pascale, was the most productive author in this field, publishing 312 articles and being cited 10,756 times in general, followed by F Stephen Hodi, the director of the Melanoma Center and Center for Immuno-Oncology of the Dana-Farber/Brigham and Women’s Cancer Center, with 301 publications and 35,302 total citations, and Caroline Robert, from the Institut de Cancérologie Gustave Roussy. Five of the top 10 cited authors were from the United States, and interestingly, the top 10 most productive authors together accounted for 9.1% of the total literature, showing the dominance of the United States in this research field. It is to be noted that Georgina V Long of Australia (190 publications), Dirk Schadendorf of Germany (184 publications), and Reinhard Dummer of Switzerland (152 publications) occupied places 6, 7 and 9 in the table, which, combined with the previous findings on geographical distribution, indicate that these countries also have important research roles in the field of ICB for melanoma.

**Table 4:** Top 10 core authors by number of publications.

Rank	Authors	Organizations	Publications	Citations	H index
1	Paolo A Ascierto	National Tumour Institute, Fondazione G. Pascale (Italy)	312	10,756	64
2	F Stephen Hodi	Dana-Farber/Brigham and Women’s Cancer Center (US)	301	35,302	93
3	Caroline Robert	Institut de Cancérologie Gustave Roussy (France)	265	27,523	78
4	Jedd D Wolchok	Memorial Sloan Kettering Cancer Center (US)	252	47,787	99
5	Antoni Ribas	University of California Los Angeles (US)	233	30,109	88
6	Georgina V Long	University of Sydney (Australia)	190	13,657	67
7	Dirk Schadendorf	University Hospital Essen (German)	184	11,733	68
8	John M Kirkwood	University of Pittsburgh Medical Center (US)	159	8493	56
9	Reinhard Dummer	University of Zurich (Switzerland)	152	7641	46
10	Steven A Rosenberg	National Cancer Institute (US)	139	27,236	99

Figure 5 is an overlay visualization of the coauthorship relationships between authors. A total of 93,587 authors were analyzed, of whom 135 met the inclusion threshold. The visualization suggested that most of the top influential authors, such as Jedd D Wolchok, F Stephen Hodi, and Antoni Ribas, had close collaborations. In addition, we found the average year of publication for these authors was between 2015 and 2016, while other authors, such as Georgina V Long, published more actively after 2018, which may suggest that they have the potential to take the lead in the future.

### Analysis of Keywords and Burst Terms

**Figure 6:**
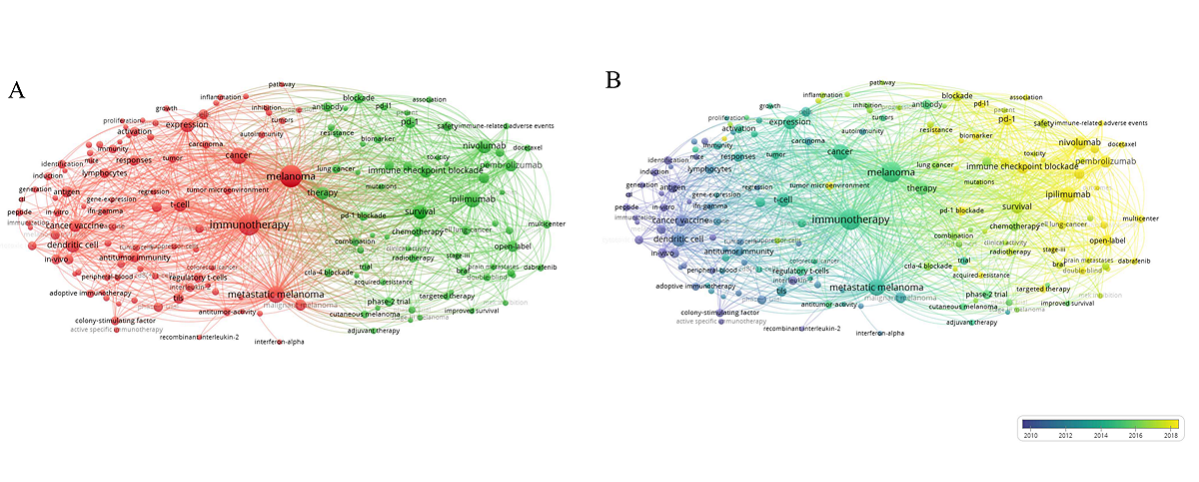
Co-occurrence analysis of keywords. These two plots show the co-occurrence of keywords. The normalization method we chose was Linlog/modularity. The weight was occurrence for each plot. (A) shows the 180 top-occurring items among 30,486 keywords, grouped into 2 clusters, with the colors of the circles representing each cluster. (B) shows the keywords grouped by year of publication, with the colors of the circles representing the average year.

In total, 30,486 keywords were extracted from 24,086 documents after removing duplicates. We used a network and overlay visualization of author-given keywords to analyze the co-occurrence of keywords. A total of 30,486 keywords were analyzed, of which the 180 most frequently occurring that met the inclusion threshold were grouped into 2 clusters (Figure 6A) and grouped by date of publication (between 2016 and 2018) (Figure 6B). The date range for publication was chosen to correspond to the high-growth phase of publication seen in Figure 2. For the network map, the keywords were mainly distinguished into 2 clusters. One mainly represented keywords related to tumor biology and the early, discovery stages of research into immunotherapy as a potentially promising modality for melanoma. These keywords are shown in red and include “antigen,” “dendritic cells,” “T cells,” “tumor microenvironment,” “immunotherapy,” “immune checkpoint,” “melanoma,” and “metastatic melanoma.” The other cluster, shown in green, generally included terms related to clinical oncology issues, including the names of US Food and Drug Administration (FDA)-proven antibodies, such as “nivolumab,” “pembrolizumab,” “atezolizumab,” and “ipilimumab”; words related to outcomes, such as “efficacy,” “safety,” and “adverse event”; and keywords that appear frequently in phase II and phase III trials, such as “survival,” ”safety,” “double-blind,” “open-label,” and “multi-center.” Interestingly, this cluster included nearly all the keywords related to targeted therapies and radiotherapy, such as “BRAF,” “MEK,” “vemurafenib,” and “dabrafenib,” as well as the names of other types of cancer, the novel usages of immunotherapy words, such as “combination” and “adjuvant therapy,” and multiple indicators for evaluating efficacy and safety. It might be the case that these interventions are now establishing more links with immunotherapy and will potentially become research hot spots in the future. The overlay map of keywords grouped by date of publication showed that research hot spots changed over time, starting with “target therapy” and moving on to “CTLA-4 inhibitor,” “PD-1/PD-L1 inhibitor,” and “FDA-proven ICB,” which is similar to the order in which these technologies developed.

**Figure 7:**
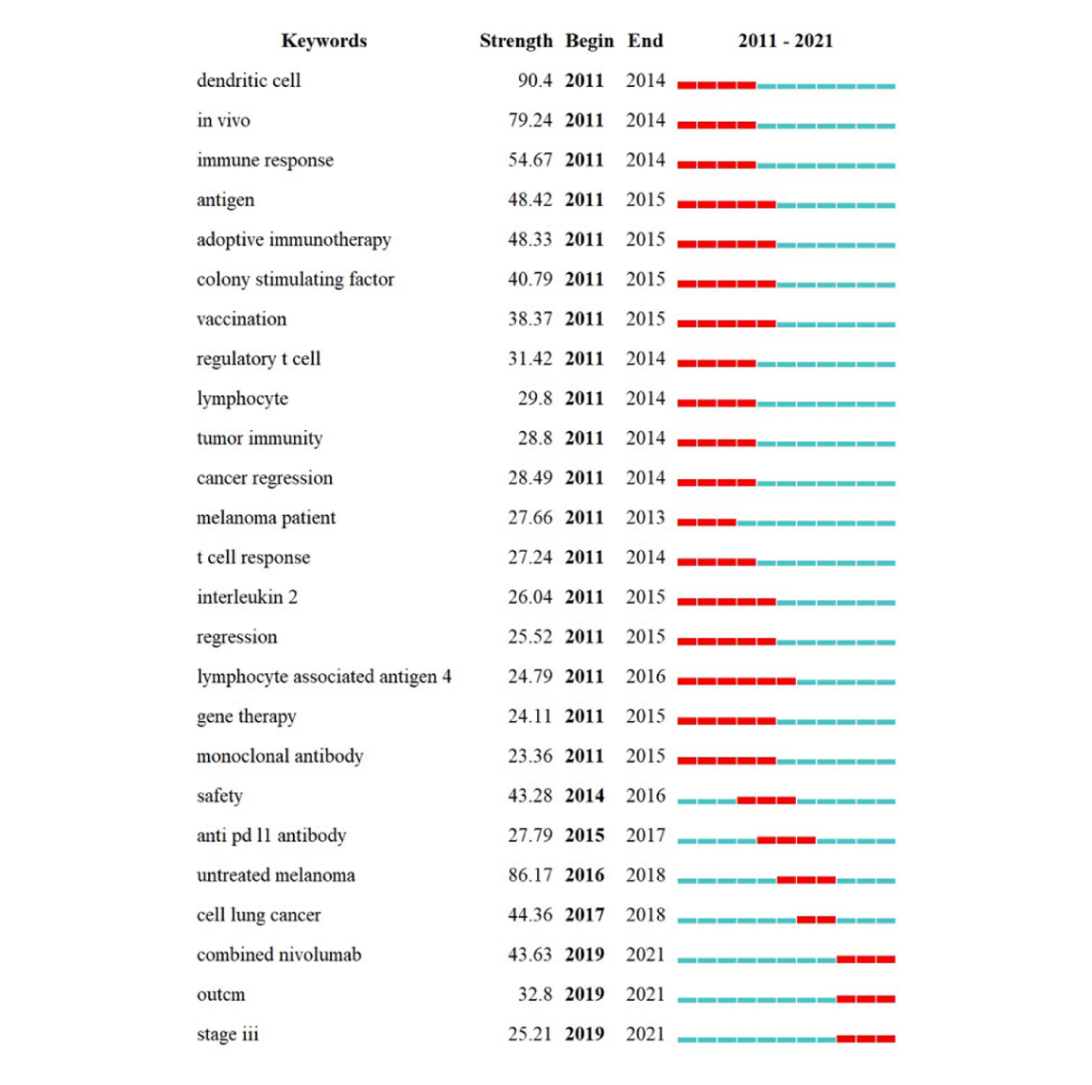
Top 25 keywords with the strongest frequency bursts. A strong frequency burst indicates that a variable has undergone a great change in a short period of time. The red bars indicate the durations of the bursts.

Figure 7 shows the top 25 keywords with the strongest frequency burst, which suggests a keyword that has undergone a great change in a short period of time (as analyzed with CiteSpace). “Dendritic cell,” “in vivo” and other mechanism- and trial-related keywords had rather stronger strength, which continued from 2011 to 2015, suggesting that possible mechanisms and clinical applications were a sustained research hot spot for these years. However, in the most recent 5 months, the burst keywords changed to include terms such as “safety,” “combined therapy,” and “stage III trial”, suggesting that the interests of the researchers changed.

### Citation Analysis

The number of citations of the publications was mainly extracted with bibliometrix. The top 10 most highly cited documents were extracted and are listed in Table 5. Generally, the number of citations ranged from 3037 to 9113. An article published in the *New England Journal of Medicine* titled “Improved survival with ipilimumab in patients with metastatic melanoma” ranked first, with 9113 total citations. Only one of the top 10 articles was a review (“The Blockade of Immune Checkpoints in Cancer Immunotherapy,” published in *Nature Reviews Cancer* in 2012). It is likely that this review was cited so often because it was the first comprehensive review of this topic. A total of 7 of the 9 articles were published in the *New England Journal of Medicine*. This shows the dominant position of this journal in the publication of research in the medical category, especially in the publication of high-quality research.

**Table 5:** Top 10 most highly cited publications.

Rank	Title	DOI^a^	Source	Publication date	Total citations^b^
1	Improved Survival with Ipilimumab in Patients with Metastatic Melanoma [10]	10.1056/NEJMoa1003466	*New Engl J Med*	Aug 2010	9549
2	Safety, Activity, and Immune Correlates of Anti-Pd-1 Antibody in Cancer [11]	10.1056/NEJMoa1200690	*New Engl J Med*	Jun 2012	7926
3	The Blockade of Immune Checkpoints in Cancer Immunotherapy [12]	10.1038/nrc3239	*Nat Rev Cancer*	Apr 2012	7160
4	Safety and Activity of Anti-Pd-L1 Antibody in Patients with Advanced Cancer [13]	10.1056/NEJMoa1200694	*New Engl J Med*	Jun 2012	5026
5	Pembrolizumab Versus Chemotherapy for Pd-L1-Positive Non-Small-Cell Lung Cancer [14]	10.1056/NEJMoa1606774	*New Engl J Med*	Nov 2016	4794
6	Combined Nivolumab and Ipilimumab or Monotherapy in Untreated Melanoma [15]	10.1056/NEJMoa1504030	*New Engl J Med*	Sep 2015	4751
7	PD-1 Blockade Induces Responses by Inhibiting Adaptive Immune Resistance [16]	10.1038/nature13954	*Nature*	Nov 2014	3514
8	Nivolumab in Previously Untreated Melanoma Without Braf Mutation [17]	10.1056/NEJMoa1412082	*New Engl J Med*	Jan 2015	3421
9	Pembrolizumab Versus Ipilimumab in Advanced Melanoma [18]	10.1056/NEJMoa1503093	*New Engl J Med*	Jun 2015	3376
10	Predictive Correlates of Response to the Anti-Pd-L1 Antibody Mpdl3280a in Cancer Patients	10.1038/nature14011	*Nature*	Nov 2014	3087

^a^DOI: Digital Object Identifier.

^b^Total citations were until the end of December 2021.

**Figure 8:**
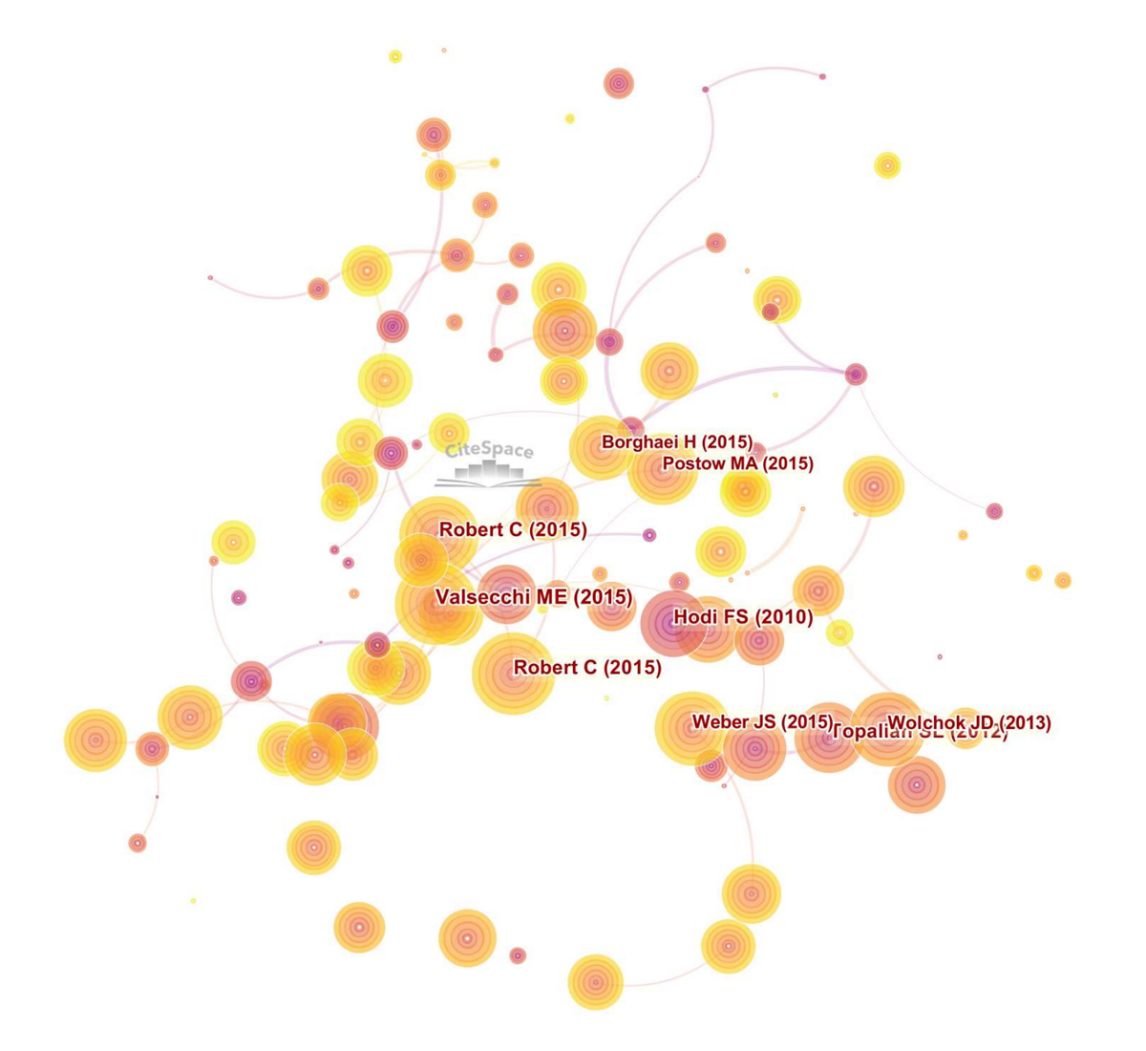
Cocitation analysis of references. Using CiteSpace, we performed a cocitation analysis of references from 2010 to 2020. In CiteSpace, the size of a circle indicates the number of documents cited. The purple area of the circle indicates the centrality of a document.

For a comprehensive analysis of citations, we used CiteSpace (version 5.8R3) to evaluate cocitation references (Figure 8). we performed a cocitation analysis of references from 2010 to 2020. In CiteSpace, the size of a circle indicates the number of documents cited. The purple area of the circle indicates the centrality of a document. The analysis revealed no significant centrality in the documents, indicating that the literature was largely scattered.

## Discussion

### Principal Findings

This study updates current knowledge on research interests related to ICB for melanoma, providing researchers and physicians an overview of the landscape of the field and potential future research hot spots. We conducted a comprehensive search of literature published on this topic before December 2021 in the Web of Science Core Collection. We retrieved 24,086 bibliographies and performed a bibliometric analysis.

First, using the bibliometric method, we analyzed chronological trends in the publications. The results show that from 1999 to 2013, the annual number of publications was rather small, with a small, linear slope of growth. The number of newly published papers from 1999 to 2013 remained under 200, with rather small growth every year. The next period was from 2014 to 2016, when publications related to ICB grew rapidly. The annual number of publications grew to over 1000, but did not reach 2000, which is consistent with previous research [19]. The third period was from 2017 to 2021, when the annual number of publications grew to over 2000, representing maturity in the theoretical aspect of this field. The number of publications has continued to increase, and the topic has gradually become a hot spot.

The chronological trend was reflected in several critical articles and specific time points, which enables us to reveal the roadmap for this field. The very first research on ICB was on CTLA-4 blockade, which was conduced beginning in 1987 and was first proved in 2011 [20,21]. The recombinant human immunoglobulin G1 and G2 monoclonal antibodies of CTLA-4, known as ipilimumab and tremelimumab, respectively, were trialed in melanoma and other advanced cancers, and both showed good efficacy [10,22-31]. As for PD-1 and PD-L1, since the very first paper on PD-1 was published in 1991, the annual number of publications remained less than 5 for a long period, from 1999 to 2003. The annual number of publications did not reach 300 until a breakthrough on cancer immunotherapy in 2013 [32]. After that, the FDA approved anti-PD-1 antibodies (pembrolizumab and nivolumab) for advanced metastatic melanoma in 2014 and an anti-PD-L1 antibody (atezolizumab) in 2016. After that, the number of publications rapidly increased, reaching the thousands [33-35]. Our results for chronological distribution, unsurprisingly, suggest that the topic of ICB for melanoma has gradually become a research hot spot, and that it is currently in a major development period. Theory developed rapidly during this period, and the number of papers increased rapidly. Moreover, the growth curve was sharp and the gradient of the curve did not slow down during 2020. We predict that this field will continue to develop in the next few years.

As for geographical distribution, among the 80 different countries and regions involved in this bibliometric analysis, the most prolific are listed in Table 2. The United States published most of the documents in this field, and Table 5 indicates that nearly all core documents were produced in partnership with American institutions [10-19]. China is the second most prolific country, but considering the number of citations, China is still far from taking the lead in this area. Other countries with a high number of publications, such as Japan, Australia, and various European counties, have a good scientific base in this field. The situation is also reflected in the most productive organizations and the most productive authors; 9 of the 10 most productive organizations and 6 of the 10 most productive authors are from the United States, which has proven the dominance of the United States in this research field.

For the most productive journals, considering the evidence for the total number of publications and IF, the *Journal of Clinical Oncology* might be the most influential journal in the topic of immune checkpoint inhibitors in melanoma. Papers published by the *Journal of Clinical Oncology* in 2021 included several that mainly concentrated on the long-term outcomes of ICB [36], the combination of ICB and other therapies, and expansion of the indications for ICB [37,38]. We also compared the IF in 2020 and 2021 in core journals in the field of ICB for melanoma and found that the IF of all top 10 core journals grew. However, considering that oncology journals generally had increased IFs in these years, we cannot make the conclusion that all journals increased their impact.

In the aspect of cooperation between authors, the network analysis showed that most cooperation took place within countries, and that there was little cooperation between countries. The same phenomenon was also revealed by a coauthorship analysis of organizations, in which clusters showed intricate connections within countries and lesser connections between clusters. These findings suggest that cooperation between states represents an area that should be strengthened.

We also performed analysis of keywords and burst terms to investigate research trends, finding that the change in focus was remarkable. Generally, research trends and the public interest changed in two major aspects: from the laboratory to translational medicine and clinical research, as well as from early ICB developments, such as CTLA-4, to later ones, such as PD-1 and PD-L1 blockades. The focus of research gradually changed from mechanisms to efficacy and adverse events. This indicates that the theory was becoming mature and that the application of ICB therapy was being explored, including enhancing its efficacy, reducing its adverse effects, and expanding its use to other, more specific cancer types [39].

From the initial research on the mechanisms of immunotherapy, including the alteration of immune cells and immune molecules under ICB treatment to subsequent translational, clinical research into the interactions of immune checkpoints with costimulatory molecules, cancer drive genes, and cancer hallmarks, studies investigating the mechanism of ICB have been maturing. In the next several years, screening of biomarkers to predict treatment efficacy and adverse events, improve the efficacy of ICB and reduce adverse events, explore drug combinations, and extend the indications for ICB might become hot spots in this clearly evolving field of research.

### Strengths and Limitations

As far as we know, this is the first study to use a bibliometric analysis to investigate research trends and public interest in ICB for melanoma. Our bibliometric analysis was much more comprehensive and intuitive than a literature review would have been, because of our use of systematic searching and quantitative statistical analysis. Moreover, we used not only CiteSpace, but also VOSviewer and the R package bibliometrix for better data extraction, bibliometric analysis, and visualization. However, this study still has some limitations. We only extracted literature from the Web of Science Core Collection database, and although this approach left little possibility for ignoring some of the documents, this type of literature might have had fewer citations. Furthermore, the bibliometric analysis methods we used can only be applied to general information, rather than full texts. Thus, we might have lost important information that only existed in the full text of the articles, such as the authors’ points of view and their prospective opinions of the field.

### Conclusion

Our bibliometric analysis should help researchers to understand the trends and public interest in ICB for melanoma. The annual number of publications was rather small, without obvious research trends at the beginning of this century, but has gradually matured in the past 6 years. In the past 2 decades, the United States has contributed the most to this field, followed by China and Germany. The top 3 most productive journals were the *Journal of Clinical Oncology*, *Cancer Immunology*, and *Immunotherapy and Cancer Research*. Cooperation between authors and organizations from different countries needs to be strengthened. In summary, ICB for melanoma is a prolific, fast-growing, and high-profile topic and more research is expected to refine knowledge in this field.

## Appendix

Multimedia Appendix 1 Comprehensive searching strategy.

## Abbreviations

CTLA-4: T-lymphocyte associated protein-4
FDA: US Food and Drug Administration
ICB: immune checkpoint blockades
IF: impact factor
PD-1: programmed death receptor 1
PD-L1: ligands of programmed death receptor 1

## References

[1] Henley SJ, Ward EM, Scott S, Ma J, Anderson RN, Firth AU, Thomas CC, Islami F, Weir HK, Lewis DR, Sherman RL, Wu M, Benard VB, Richardson LC, Jemal A, Cronin K, Kohler BA (2020). Annual report to the nation on the status of cancer, part I: National cancer statistics. Cancer.

[2] Fitzmaurice C (2018). Global, regional, and national cancer incidence, mortality, years of life lost, years lived with disability, and disability-adjusted life-years for 29 cancer groups, 2006 to 2016: A systematic analysis for the Global Burden of Disease study. JCO.

[3] Skin cancer statistics. World Cancer Research Fund International.

[4] Robbins HA, Clarke CA, Arron ST, Tatalovich Z, Kahn AR, Hernandez BY, Paddock L, Yanik EL, Lynch CF, Kasiske BL, Snyder J, Engels EA (2015). Melanoma Risk and Survival among Organ Transplant Recipients. J Invest Dermatol.

[5] Shiels MS, Copeland G, Goodman MT, Harrell J, Lynch CF, Pawlish K, Pfeiffer RM, Engels EA (2015). Cancer stage at diagnosis in patients infected with the human immunodeficiency virus and transplant recipients. Cancer.

[6] Alexandrov LB, Nik-Zainal S, Wedge DC, Aparicio SAJR, Behjati S, Biankin AV, Bignell GR, Bolli N, Borg A, Børresen-Dale Anne-Lise, Boyault S, Burkhardt B, Butler AP, Caldas C, Davies HR, Desmedt C, Eils R, Eyfjörd Jórunn Erla, Foekens JA, Greaves M, Hosoda F, Hutter B, Ilicic T, Imbeaud S, Imielinski M, Imielinsk M, Jäger Natalie, Jones DTW, Jones D, Knappskog S, Kool M, Lakhani SR, López-Otín Carlos, Martin S, Munshi NC, Nakamura H, Northcott PA, Pajic M, Papaemmanuil E, Paradiso A, Pearson JV, Puente XS, Raine K, Ramakrishna M, Richardson AL, Richter J, Rosenstiel P, Schlesner M, Schumacher TN, Span PN, Teague JW, Totoki Y, Tutt ANJ, Valdés-Mas Rafael, van Buuren MM, van 't Veer L, Vincent-Salomon A, Waddell N, Yates LR, Zucman-Rossi J, Futreal PA, McDermott U, Lichter P, Meyerson M, Grimmond SM, Siebert R, Campo E, Shibata T, Pfister SM, Campbell PJ, Stratton MR, Australian Pancreatic Cancer Genome Initiative, ICGC Breast Cancer Consortium, ICGC MMML-Seq Consortium, ICGC PedBrain (2013). Signatures of mutational processes in human cancer. Nature.

[7] Cooper ID (2015). Bibliometrics basics. J Med Libr Assoc.

[8] Pendlebury DA White paper: Using bibliometrics in evaluating research. Research Department, Thomson Reuters.

[9] Taylor BE, McClave SA, Martindale RG, Warren MM, Johnson DR, Braunschweig C, McCarthy MS, Davanos E, Rice TW, Cresci GA, Gervasio JM, Sacks GS, Roberts PR, Compher C (2016). Guidelines for the Provision and Assessment of Nutrition Support Therapy in the Adult Critically Ill Patient. Crit Care Med.

[10] Hodi FS, O'Day SJ, McDermott DF, Weber RW, Sosman JA, Haanen JB, Gonzalez R, Robert C, Schadendorf D, Hassel JC, Akerley JM, van den Eertwegh AJ, Lutzky J, Lorigan P, Vaubel JM, Linette GP, Hogg D, Ottensmeier CH, Lebbé C, Peschel C, Quirt I, Clark JI, Wolchok JD, Weber JS, Tian J, Yellin MJ, Nichol GM, Hoos A, Urba WJ (2010). Improved survival with ipilimumab in patients with metastatic melanoma. N Engl J Med.

[11] Topalian SL, Hodi FS, Brahmer JR, Gettinger SN, Smith DC, McDermott DF, Powderly JD, Carvajal RD, Sosman JA, Atkins MB, Leming PD, Spigel DR, Antonia SJ, Horn L, Drake CG, Pardoll DM, Chen L, Sharfman WH, Anders RA, Taube JM, McMiller TL, Xu H, Korman AJ, Jure-Kunkel M, Agrawal S, McDonald D, Kollia GD, Gupta A, Wigginton JM, Sznol M (2012). Safety, activity, and immune correlates of anti-PD-1 antibody in cancer. N Engl J Med.

[12] Pardoll DM (2012). The blockade of immune checkpoints in cancer immunotherapy. Nat Rev Cancer.

[13] Brahmer JR, Tykodi SS, Chow LQ, Hwu W, Topalian SL, Hwu P, Drake CG, Camacho LH, Kauh J, Odunsi K, Pitot HC, Hamid O, Bhatia S, Martins R, Eaton K, Chen S, Salay TM, Alaparthy S, Grosso JF, Korman AJ, Parker SM, Agrawal S, Goldberg SM, Pardoll DM, Gupta A, Wigginton JM (2012). Safety and activity of anti-PD-L1 antibody in patients with advanced cancer. N Engl J Med.

[14] Reck M, Rodríguez-Abreu Delvys, Robinson AG, Hui R, Csőszi T, Fülöp Andrea, Gottfried M, Peled N, Tafreshi A, Cuffe S, O'Brien Mary, Rao S, Hotta K, Leiby MA, Lubiniecki GM, Shentu Y, Rangwala R, Brahmer JR, KEYNOTE-024 Investigators (2016). Pembrolizumab versus Chemotherapy for PD-L1-Positive Non-Small-Cell Lung Cancer. N Engl J Med.

[15] Larkin J, Hodi FS, Wolchok JD (2015). Combined Nivolumab and Ipilimumab or Monotherapy in Untreated Melanoma. N Engl J Med.

[16] Tumeh PC, Harview CL, Yearley JH, Shintaku IP, Taylor EJM, Robert L, Chmielowski B, Spasic M, Henry G, Ciobanu V, West AN, Carmona M, Kivork C, Seja E, Cherry G, Gutierrez AJ, Grogan TR, Mateus C, Tomasic G, Glaspy JA, Emerson RO, Robins H, Pierce RH, Elashoff DA, Robert C, Ribas A (2014). PD-1 blockade induces responses by inhibiting adaptive immune resistance. Nature.

[17] Robert C, Long GV, Brady B, Dutriaux C, Maio M, Mortier L, Hassel JC, Rutkowski P, McNeil C, Kalinka-Warzocha E, Savage KJ, Hernberg MM, Lebbé C, Charles J, Mihalcioiu C, Chiarion-Sileni V, Mauch C, Cognetti F, Arance A, Schmidt H, Schadendorf D, Gogas H, Lundgren-Eriksson L, Horak C, Sharkey B, Waxman IM, Atkinson V, Ascierto PA (2015). Nivolumab in Previously Untreated Melanoma without Mutation. N Engl J Med.

[18] Robert C, Schachter J, Long GV, Arance A, Grob JJ, Mortier L, Daud A, Carlino MS, McNeil C, Lotem M, Larkin J, Lorigan P, Neyns B, Blank CU, Hamid O, Mateus C, Shapira-Frommer R, Kosh M, Zhou H, Ibrahim N, Ebbinghaus S, Ribas A, KEYNOTE-006 investigators (2015). Pembrolizumab versus Ipilimumab in Advanced Melanoma. N Engl J Med.

[19] Gao Y, Shi S, Ma W, Chen J, Cai Y, Ge L, Li L, Wu J, Tian J (2019). Bibliometric analysis of global research on PD-1 and PD-L1 in the field of cancer. Int Immunopharmacol.

[20] Brunet J, Denizot F, Luciani M, Roux-Dosseto M, Suzan M, Mattei M, Golstein P (1987). A new member of the immunoglobulin superfamily--CTLA-4. Nature.

[21] Sharma P, Allison JP (2015). The future of immune checkpoint therapy. Science.

[22] Hodi F, Mihm Martin C, Soiffer Robert J, Haluska Frank G, Butler Marcus, Seiden Michael V, Davis Thomas, Henry-Spires Rochele, MacRae Suzanne, Willman Ann, Padera Robert, Jaklitsch Michael T, Shankar Sridhar, Chen Teresa C, Korman Alan, Allison James P, Dranoff Glenn (2003). Biologic activity of cytotoxic T lymphocyte-associated antigen 4 antibody blockade in previously vaccinated metastatic melanoma and ovarian carcinoma patients. Proc Natl Acad Sci U S A.

[23] Phan GQ, Yang JC, Sherry RM, Hwu P, Topalian SL, Schwartzentruber DJ, Restifo NP, Haworth LR, Seipp CA, Freezer LJ, Morton KE, Mavroukakis SA, Duray PH, Steinberg SM, Allison JP, Davis TA, Rosenberg SA (2003). Cancer regression and autoimmunity induced by cytotoxic T lymphocyte-associated antigen 4 blockade in patients with metastatic melanoma. Proc Natl Acad Sci U S A.

[24] Attia P, Phan GQ, Maker AV, Robinson MR, Quezado MM, Yang JC, Sherry RM, Topalian SL, Kammula US, Royal RE, Restifo NP, Haworth LR, Levy C, Mavroukakis SA, Nichol G, Yellin MJ, Rosenberg SA (2005). Autoimmunity Correlates With Tumor Regression in Patients With Metastatic Melanoma Treated With Anti–Cytotoxic T-Lymphocyte Antigen-4. JCO.

[25] Sanderson K, Scotland R, Lee P, Liu D, Groshen S, Snively J, Sian S, Nichol G, Davis T, Keler T, Yellin M, Weber J (2005). Autoimmunity in a phase I trial of a fully human anti-cytotoxic T-lymphocyte antigen-4 monoclonal antibody with multiple melanoma peptides and Montanide ISA 51 for patients with resected stages III and IV melanoma. J Clin Oncol.

[26] Comin-Anduix B, Lee Y, Jalil J, Algazi A, de la Rocha P, Camacho LH, Bozon VA, Bulanhagui CA, Seja E, Villanueva A, Straatsma BR, Gualberto A, Economou JS, Glaspy JA, Gomez-Navarro J, Ribas A (2008). Detailed analysis of immunologic effects of the cytotoxic T lymphocyte-associated antigen 4-blocking monoclonal antibody tremelimumab in peripheral blood of patients with melanoma. J Transl Med.

[27] Ménard Cédric, Ghiringhelli François, Roux Stephan, Chaput Nathalie, Mateus Christine, Grohmann Ursula, Caillat-Zucman Sophie, Zitvogel Laurence, Robert Caroline (2008). Ctla-4 blockade confers lymphocyte resistance to regulatory T-cells in advanced melanoma: surrogate marker of efficacy of tremelimumab?. Clin Cancer Res.

[28] Ribas A, Comin-Anduix Begoña, Economou James S, Donahue Timothy R, de la Rocha Pilar, Morris Lilah F, Jalil Jason, Dissette Vivian B, Shintaku Itsushi Peter, Glaspy John A, Gomez-Navarro Jesus, Cochran Alistair J (2009). Intratumoral immune cell infiltrates, FoxP3, and indoleamine 2,3-dioxygenase in patients with melanoma undergoing CTLA4 blockade. Clin Cancer Res.

[29] Vonderheide R, LoRusso Patricia M, Khalil Magi, Gartner Elaina M, Khaira Divis, Soulieres Denis, Dorazio Prudence, Trosko Jennifer A, Rüter Jens, Mariani Gabriella L, Usari Tiziana, Domchek Susan M (2010). Tremelimumab in combination with exemestane in patients with advanced breast cancer and treatment-associated modulation of inducible costimulator expression on patient T cells. Clin Cancer Res.

[30] Lynch Thomas J, Bondarenko Igor, Luft Alexander, Serwatowski Piotr, Barlesi Fabrice, Chacko Raju, Sebastian Martin, Neal Joel, Lu Haolan, Cuillerot Jean-Marie, Reck Martin (2012). Ipilimumab in combination with paclitaxel and carboplatin as first-line treatment in stage IIIB/IV non-small-cell lung cancer: results from a randomized, double-blind, multicenter phase II study. J Clin Oncol.

[31] Slovin S, Higano C, Hamid O, Tejwani S, Harzstark A, Alumkal J, Scher H, Chin K, Gagnier P, McHenry M, Beer T (2013). Ipilimumab alone or in combination with radiotherapy in metastatic castration-resistant prostate cancer: results from an open-label, multicenter phase I/II study. Ann Oncol.

[32] Couzin-Frankel J (2013). Breakthrough of the year 2013. Cancer immunotherapy. Science.

[33] Poole RM (2014). Pembrolizumab: first global approval. Drugs.

[34] Wang J, Yuan R, Song W, Sun J, Liu D, Li Z (2017). PD-1, PD-L1 (B7-H1) and Tumor-Site Immune Modulation Therapy: The Historical Perspective. J Hematol Oncol.

[35] Markham A (2016). Atezolizumab: First Global Approval. Drugs.

[36] Wolchok Jedd D, Chiarion-Sileni Vanna, Gonzalez Rene, Grob Jean-Jacques, Rutkowski Piotr, Lao Christopher D, Cowey C Lance, Schadendorf Dirk, Wagstaff John, Dummer Reinhard, Ferrucci Pier Francesco, Smylie Michael, Butler Marcus O, Hill Andrew, Márquez-Rodas Ivan, Haanen John B A G, Guidoboni Massimo, Maio Michele, Schöffski Patrick, Carlino Matteo S, Lebbé Céleste, McArthur Grant, Ascierto Paolo A, Daniels Gregory A, Long Georgina V, Bas Tuba, Ritchings Corey, Larkin James, Hodi F Stephen (2022). Long-Term Outcomes With Nivolumab Plus Ipilimumab or Nivolumab Alone Versus Ipilimumab in Patients With Advanced Melanoma. J Clin Oncol.

[37] Olson DJ, Eroglu Z, Brockstein B, Poklepovic AS, Bajaj M, Babu S, Hallmeyer S, Velasco M, Lutzky J, Higgs E, Bao R, Carll TC, Labadie B, Krausz T, Zha Y, Karrison T, Sondak VK, Gajewski TF, Khushalani NI, Luke JJ (2021). Pembrolizumab Plus Ipilimumab Following Anti-PD-1/L1 Failure in Melanoma. J Clin Oncol.

[38] Pelster MS, Gruschkus SK, Bassett R, Gombos DS, Shephard M, Posada L, Glover MS, Simien R, Diab A, Hwu P, Carter BW, Patel SP (2021). Nivolumab and Ipilimumab in Metastatic Uveal Melanoma: Results From a Single-Arm Phase II Study. J Clin Oncol.

[39] Waldman AD, Fritz JM, Lenardo MJ (2020). A guide to cancer immunotherapy: from T cell basic science to clinical practice. Nat Rev Immunol.

