# Supplementary material for: Research Trends in Immune Checkpoint Blockade for Melanoma: Visualization and Bibliometric Analysis
Source: J Med Internet Res. 2022 Jun 27;24(6):e32728. doi: 10.2196/32728 (PMC9274394; doi:10.2196/32728)
Supplement: Multimedia Appendix 1 [file jmir_v24i6e32728_app1.docx]

Immune checkpoint blockade search term:

| 1 | TS=(ipilimumab) OR TI=(ipilimumab) OR AB=(ipilimumab) |
| --- | --- |
| 2 | TS=(pembrolizumab) OR TI=(pembrolizumab) OR AB=(pembrolizumab) |
| 3 | TS=(nivolumab) OR TI=(nivolumab) OR AB=(nivolumab) |
| 4 | TS=(immunotherapy) OR TI=(immunotherapy) OR AB=(immunotherapy) OR TS=(immune checkpoint blockade) OR TI=(immune checkpoint blockade) OR AB=(immune checkpoint blockade) OR TS=(immune checkpoint inhibitor) OR TI=(immune checkpoint inhibitor) OR AB=(immune checkpoint inhibitor) |
| 5 | TS=(PD-1) OR TI=(PD-1) OR AB=(PD-1) OR TS=(PD-L1) OR TI=(PD-L1) OR AB=(PD-L1) OR TS=(CTLA-4) OR TI=(CTLA-4) OR AB=(CTLA-4) |
| 6 | TI=(yervoy) OR AB=(yervoy) OR TI=(keytruda) OR AB=(keytruda) OR TI=(opdivo) OR AB=(opdivo) |
| 7 | #1 OR #2 OR #3 OR #4 OR #5 OR #6 |

Melanoma search term:

| 8 | TI=(melano*) OR AB=(melano*) |
| --- | --- |
| 9 | TS=(melanoma) OR TI=(melanoma) OR AB=(melanoma) |
| 10 | TS=(melanocarcinoma) OR TI=(melanocytoma) |
| 11 | #8 OR #9 OR #10 |

#7 AND #11

Time: 1999-2021.

Database: WOS core.

Language = English
